# Host-dependent alteration of the gut microbiota: the role of luminal microRNAs

**DOI:** 10.20517/mrr.2024.46

**Published:** 2025-02-22

**Authors:** Céline Cuinat, Jiali Pan, Elena M. Comelli

**Affiliations:** ^1^Department of Nutritional Sciences, Faculty of Medicine, University of Toronto, Toronto M5S 1A8, Canada.; ^2^Joannah and Brian Lawson Centre for Child Nutrition, Faculty of Medicine, University of Toronto, Toronto M5S 1A8, Canada.; ^#^Authors contributed equally.

**Keywords:** MicroRNA, gut microbiota, regulatory RNA, intestine, fecal, miRNome, probiotics, dietary microRNA

## Abstract

MicroRNAs (miRNAs) are short, non-coding RNAs that play gene expression regulatory roles in eukaryotes. MiRNAs are also released in body fluids, and in the intestine, they are found in the lumen and feces. Here, together with exogenous dietary-derived miRNAs, they constitute the fecal miRNome. Several miRNAs were identified in the feces of healthy adults, including, as shown here, core miRNAs hsa-miR-21-5p and hsa-miR-1246. These miRNAs are important for intestinal homeostasis. Recent evidence suggests that miRNAs may interact with gut bacteria. This represents a new avenue to understand host-bacteria crosstalk in the gut and its role in health and disease. This review provides a comprehensive overview of current knowledge on fecal miRNAs, their representation across individuals, and their effects on the gut microbiota. It also discusses existing evidence on potential mechanisms of uptake and interaction with bacterial genomes, drawing from knowledge of prokaryotic small RNAs (sRNAs) regulation of gene expression. Finally, we review *in silico* and experimental approaches for profiling miRNA-mRNA interactions in bacterial species, highlighting challenges in target validation. This work emphasizes the need for further research into host miRNA-bacterial interactions to better understand their regulatory roles in the gut ecosystem and support their exploitation for disease prevention and treatment.

## INTRODUCTION

The intestinal ecosystem relies on continuous communication between members of the resident microbial community and host cells. The diversity and complexity of the interactions allow for immediate and long-term responses to environmental (luminal) stimuli, as well as for their fine-tuning and specificity. These processes are essential for maintaining host health and recovering from disease states. The bidirectional aspect of these interactions is critical, and interestingly, host and microbial cells share strategies to affect each other. These include the release of metabolites^[1,2]^, peptides^[3,4]^, hormone-like substances^[5,6]^, and receptor-mediated or independent responses that result in gene expression regulation.

Eukaryotic microRNAs (miRNAs) have emerged as a novel host-dependent mechanism that affects the gut microbiota^[7]^. MiRNAs are single-stranded, non-coding RNAs of about 22 nucleotides in length. They regulate gene expression post-transcriptionally by either binding to their target mRNAs to inhibit translation or promoting their degradation^[8]^. A total of 2,656 mature miRNAs have been identified in humans^[9]^. MiRNA-mediated gene expression regulation appears to be an evolutionarily conserved mechanism since it has been found in both multicellular and unicellular organisms^[10]^. In addition, select miRNAs (for example, miR-21) have high sequence similarity across multiple species. Therefore, these miRNAs could potentially play roles in interspecies crosstalk regulation. In humans, miRNAs regulate over 60% of the protein-coding genes^[11]^. Intestinal miRNAs are involved in several processes, including cell growth, differentiation, development, apoptosis, immune response, and metabolism^[12]^. Knockout of Dicer, the enzyme responsible for the generation of mature miRNA transcripts, results in disrupted mucosal architecture^[13]^ and propensity to inflammation^[14]^. MiRNA expression is regulated either by transcriptional mechanisms, such as DNA methylation and transcription factors or by post-transcriptional mechanisms, such as primary (pri-) and precursor (pre-) miRNA processing and miRNA degradation^[15]^. In the intestine, the expression of mature miRNAs also depends on the gut microbiota. Studies in germ-free animals showed that the presence of the microbial community affects the expression of miRNAs involved in several processes in both the small and large intestine^[16,17]^, including permeability, angiogenesis, and immune response. In addition, microbiota modulation by pathogenic or beneficial bacteria in the gut affects host miRNA expression profiles^[18-21]^, showing that the composition of the microbiota is also important. Microbial strategies mediating these microbiota-dependent effects encompass direct interaction between bacteria and host cells^[22]^ and bacterial products such as lipopolysaccharide^[23]^, metabolites (butyrate^[24]^), and genotoxins (colibactin^[25]^). For example, butyrate administration reduces c-myc expression in human colon cancer cells and, in turn, the abundance of miR-92a in these cells^[24]^.

MiRNAs are known to be released by eukaryotic cells and function as messengers between adjacent or distal cells^[26,27]^. In line with this, miRNAs can be recovered in body fluids, such as blood^[28]^, urine^[29]^, saliva^[30]^, and feces^[31]^. MiRNAs in feces (generally referred to as fecal miRNAs) resist degradation^[32]^ and their concentration is stable over several months in healthy conditions^[33]^. Several miRNA species have been identified in human fecal matter, though it is unknown whether a core set of fecal miRNAs exists that is shared among individuals. This is important because fecal miRNAs have been proposed as non-invasive biomarkers of various diseases, such as inflammatory bowel diseases^[34,35]^ and pancreatic^[36]^ and colorectal cancers^[37,38]^. In addition, aligned with their intestinal cell origin, fecal miRNAs have been proposed as markers of microbiota eubiosis. For instance, Viennois *et al.* identified a group of 12 miRNAs that are correlated with microbial taxa and function as a marker of gut microbiota healthiness and colitogenic potential^[39]^. The fecal miRNome also comprises diet-derived miRNAs that escape proximal degradation and reach distal intestinal regions. Both host- and diet-derived miRNAs were shown to affect the growth of bacterial members of the microbiota. Bacteria are not known to express *bona fide* miRNAs; thus, these findings open new intriguing research avenues on inter-kingdom gene expression regulatory mechanisms. While findings to date suggest that eukaryotic miRNAs may regulate prokaryotic genes, mechanisms of miRNA entry into bacteria and their mode of action remain elusive. In eukaryotes, the identification of miRNA gene targets has evolved from transcriptomics studies focused on the relative expression of miRNA-mRNA pairs^[40]^ to incorporate computational and machine learning approaches, which was accompanied by the development of tools allowing researchers to query across multiple databases^[41,42]^. However, there are limited bioinformatics tools and pipelines to support the understanding of miRNA function in bacteria. Here, we review current knowledge on fecal miRNAs, including their origin, diversity, and stability, and provide an assessment of their variability among individuals. We discuss evidence of their effects on microbiota gene regulation and growth, including that of both resident and allochthonous members of the microbiota. We then explore potential mechanisms underlying miRNA regulation of bacterial gene expression, building on current knowledge of prokaryotic small RNA (sRNA) systems. Finally, we discuss *in silico* approaches used to predict bacterial miRNA gene targets and discuss experimental approaches for validating these predictions.

## FECAL MIRNAs: ORIGIN AND DIVERSITY

The biogenesis of miRNAs starts in the nucleus, where miRNA genes are transcribed into pri-miRNA transcripts of several hundred nucleotides. These pri-miRNAs are then cleaved by the enzyme Drosha into shorter (~60-70 nucleotides) pre-miRNAs with a characteristic hairpin structure. Pre-miRNAs are subsequently exported to the cytoplasm, where they undergo further processing by a second endonuclease, Dicer, that cleaves the loop of the hairpin, forming short double-strand miRNA-miRNA duplexes^[43]^. The miRNA strands originating from the 5’ or the 3’ arms of the hairpin loop are named -5p and -3p, respectively^[9]^. In animals, one strand, known as the guide strand, is typically retained to regulate gene expression, as opposed to the other strand, referred to as the passenger strand, which is usually degraded within a few hours^[44]^. This is likely due to the guide strand being associated with the protein argonaute 2 (Ago2)^[44]^. Ago2 is found in the RNA-induced silencing complex (RISC), which is responsible for miRNA-mediated mRNA target binding^[45]^. The selectivity of the strand is accomplished by Ago2 preferentially binding to a strand with relative thermodynamic instability and uracil on the 5’ end^[45]^. In addition, the phosphate moiety of the 5’ nucleotide must be accessible for Ago2^[45]^. This miRNA strand selection process can be dysregulated in certain physiological or pathological conditions, such as cancer^[45]^, where the abundance of multiple miRNA passenger strands is altered. For example, in glioblastoma, the passenger strand of miR-324 is upregulated, while its guide strand is downregulated^[46]^. In addition, in squamous cell carcinoma, both the guide and passenger strands of miR-21 are upregulated^[47]^, and in lung cancer, both the guide and passenger strands of miR-144 are downregulated^[48]^. The seed sequence of a miRNA corresponds to the first 2-8 nucleotides^[49]^. In eukaryotes, this region is used to recognize the mRNA target, making it an important feature in speculating miRNA function. Mature miRNA strands incorporated into RISC partially bind complementary sequences in the 3’ untranslated region (UTR) of the target mRNA to regulate gene expression. Depending on the degree of complementarity between miRNA and mRNA sequences, a mature miRNA will either cleave its target or inhibit its translation^[50]^. MiRNAs can also target protein-coding sequences (CDS) through unusual mechanisms requiring extensive base pairings in the miRNA 3’ end^[51]^. Additionally, under specific conditions, miRNAs can upregulate gene expression by binding to the 3’ or the 5’ UTR of their target mRNA^[52,53]^.

Besides intracellular gene regulation, upon their extracellular release, miRNAs also act as messenger molecules in eukaryotic cell-to-cell communication to affect gene regulation distally. Given that a single miRNA can target multiple mRNAs, and different miRNAs can target the same mRNA, secreted miRNAs likely participate in intricate gene regulatory networks within their target cells. Both pre-miRNAs and mature miRNAs can be released from cells in small extracellular vesicles (EVs) like exosomes^[54,55]^ or transported by high-density lipoproteins (HDL)^[56]^, although naked forms have also been detected. Regardless of their mode of release, most extracellular mature miRNAs are associated with proteins such as Ago2 or nucleophosmin 1 (NPM1)^[57,58]^. The mechanisms governing which miRNAs are selected for cellular release, the ratio of mature to pre-miRNA secreted, and how they are delivered into target cells remain poorly understood. In contrast, more knowledge is available about how miRNAs enter cells. Entrance in target cells differs between vesicle-associated and vesicle-free miRNAs. It is thought that the first enter via endocytosis^[59]^, phagocytosis, or direct fusion with the cell plasma membrane. For example, bone marrow-derived dendritic cells transfer endogenous exosomes carrying multiple miRNAs, including miR-21, miR-221, and miR-222, to target dendritic cells through fusion^[60]^. On the other hand, vesicle-free miRNAs are taken up either via specific receptors^[56,61]^ or directly through gap junctions^[62]^. For example, miR-142 and miR-223 have been shown to transfer from macrophages to hepato-carcinoma cells through a mechanism dependent on cell-to-cell contact and gap junctions^[62]^. Cell-free miRNAs are very stable due to their structural characteristics, which protect them from RNA-degrading enzymes and RNase activity. This stability allows them to persist and function in extracellular environments. MiRNAs enclosed within EVs are particularly resilient, exhibiting greater stability than vesicle-free miRNAs^[63,64]^. In addition, the stability of extracellular miRNAs is correlated with their GC content, suggesting unequal stability among different miRNAs^[64]^.

In the intestine, where the apical surface of the epithelial cells faces the lumen, miRNAs can be directly released into the luminal content and referred to as luminal or fecal miRNAs. A limited number of studies have investigated the cellular origin of fecal miRNAs. A seminal study found them to derive from exfoliated colonocytes^[65]^. This is in line with our previous work, where we found correlations between mouse cecal content and tissue miRNA signatures, although partial^[21]^. Later, enterocytes were determined to be the major source of luminal miRNAs, together with homeodomain only protein (Hopx)-expressing cells such as Paneth and goblet cells^[7]^. Specifically, the abundance of 53% of 344 fecal miRNAs and 12% of 360 fecal miRNAs was reduced in mice lacking Dicer in intestinal epithelial cells or Hopx-expressing cells, respectively^[7]^. The packaging of fecal miRNAs remains under-investigated. While they have been detected in exosomes, which are abundant in human feces, it remains uncertain if they also exist in an EV-free form, such as protein-bound^[7,66]^. Similarly to other body fluids, exosomes protect miRNAs from RNase activity in feces, where naked miRNAs are more rapidly degraded^[67]^.

Exogenous sources additionally contribute to the fecal miRNome, including dietary components such as animal and plant products^[68]^. These dietary miRNAs are very stable during food harvesting and processing, including cooking^[69,70]^. Following ingestion, these miRNAs withstand digestion^[70,71]^ and the unfavorable environment of the stomach and proximal small intestine. This resistance is largely conferred by their packaging in EVs^[72]^ for animal-derived miRNAs, or exosome-like nanoparticles^[73]^ and 3’ end modification^[74]^ for plant-derived miRNAs. The availability of miRNAs in foods has been recently reviewed^[75]^ and their recovery in the intestine, and then in plasma, demonstrated. For instance, when piglets were fed bovine milk containing a reporter miRNA, this miRNA was detected in the bloodstream, which suggests that it can survive digestion and cross the intestinal barrier^[76]^. Plant-derived miRNAs, such as ath-MIR162a, were found in watermelon juice one hour after preparation and could be recovered in plasma following ingestion^[77]^. Similarly, plant MIR168a was detectable in the serum of mice six hours after fresh rice or fresh rice total RNA ingestion^[78]^. Meat miRNAs, such as miR-10b-5p and miR-206, can be found in cooked muscle tissues^[75]^. Additionally, miRNAs, such as miR-21 and miR-16, are present in various foods, including poultry, meat, egg, and cheese, although their expression levels vary between food types^[79]^. We, therefore, speculate that dietary miRNA could contribute to a transient fecal miRNome. Besides serving as a direct source of fecal miRNAs, diet can also indirectly affect their abundance through the host. Both specific nutrients and dietary components, as well as dietary patterns, may influence fecal miRNA profiles. For example, oligosaccharides^[80]^ and polyphenols^[81]^ were found to affect miRNA expression in intestinal cells, potentially altering their concentration in the lumen if released. Interestingly, a vegetarian diet has been shown to increase the presence of plant MIR168a in feces, illustrating that dietary intake can influence the abundance of specific miRNAs in fecal matter^[79]^. In addition, it was found that individuals on a vegetarian or vegan diet have a lower abundance of miR-636 and miR-4739 in their feces compared to those on an omnivorous diet^[82]^. Notably, the expression levels of these miRNAs inversely correlate with the number of years spent on the diet. In celiac disease patients, a gluten-free diet alters the levels of fecal miR-4533-3p and miR-2681-3p, which, interestingly, return to control (healthy) levels in those with longer adherence^[83]^. Thus, these findings suggest that fecal miRNAs may represent the effects of dietary changes on intestinal cell physiology^[83]^.

Eukaryotic members of the gut microbiota may represent a third source of fecal miRNAs. The fungus *Candida albicans* and the parasite *Giardia lamblia* were found to carry miRNA-size (ms)RNAs and miRNA precursors, respectively^[84,85]^, and *Giardia duodenalis* EVs were found to contain various RNA species, including miRNA^[86]^. Therefore, it is possible that eukaryotic members of the microbiota could also release miRNA in fecal matter, although this remains largely under-investigated.

Toward the understanding of the healthy human miRNome, we identified 17 studies profiling the fecal miRNAs of healthy adult humans. These studies were conducted across various countries, with samples collected from individuals of different age groups and ethnicities. Many studies explored the effects of different diets, while others focused on specific diseases. We hypothesized that a shared set of miRNAs may exist across individuals, forming a health-compatible human fecal miRNome. We used 11 miRNA datasets that were deposited by these studies in publicly available databases [Table 1], 7 of which were unique.

**Table 1:** Overview of healthy adult human fecal miRNA studies

**Participants demographics [country, age (years), female %]**	**Sample size**	**Method of miRNA assessment**	**Number of fecal miRNAs detected**	**Ref.**
United States, mean age = 23 ± 1.63, 50% female	*N* = 4	RNA sequencing and alignment using miRBase V20, 1,765,452 ± 488,850 average raw sequencing read detected, 0.2% ± 0.04% aligned to database	21	Seashols-Williams *et al.* 2016^[87]^
Italy, mean age = 44.7 ± 14.7, 63.6% female	*N* = 335 (Samples in this study include samples from^[82]^ and^[88]^)	RNA sequencing and alignment using in-house reference based on miRbase v22, 10.3 million average raw sequencing reads, 0.92% aligned to database	449	Francavilla *et al.* 2021^[33]^
Italy, omnivores, mean age = 40.5 ± 13.2, 60% female Vegetarians, mean age = 40.6 ± 11.7, 60% female Vegans, mean age = 39.1 ± 11.6, 60% female	*N* = 120 (40 omnivores, 40 vegetarians, 40 vegans)	RNA sequencing and alignment using in-house reference based on miRbase v22, 7.8 million average raw sequencing reads, 0.83% aligned to database	145	Tarallo *et al.* 2022^[82]^
Italy, adults	*N* = 39	RNA sequencing and alignment using miRbase v21, 14.66 million average raw sequencing reads, 0.7% aligned to database	102	Ferrero *et al.* 2017^[88]^
United States, age 24-59, 70% female	*N* = 10	nCounter® Human miRNA Expression Assay (NanoString Technologies)	181	Liu *et al.* 2016^[7]^
United States, mean age = 49, 75% female	Healthy control for multiple sclerosis *N* = 12	RNA sequencing and alignment using miRBase through exceRpt sRNA-seq pipeline v4.6.2^[89]^, 23.77 million average raw sequencing reads	25	Liu *et al.* 2019^[90]^
Italy, adults	Healthy control for colorectal cancer *N* = 24	RNA sequencing and alignment using in-house reference based on miRbase v21, 9.3 million average raw sequencing reads	64	Tarallo *et al.* 2019^[91]^
Italy, control, mean age = 40.8 ± 14.3, 77.3% female Validation cohort mean age 40.5 ± 13.2, 60% female	Healthy control for celiac disease *N* = 106 (66 control, 40 samples from^[82]^)	RNA sequencing and alignment using in-house reference based on miRbase v21, 11.1 million average raw sequencing reads, 1.03% aligned to database	757	Francavilla *et al.* 2023^[83]^
Italy, mean age = 59.6 ± 10.7, 50.5% female Czech Republic, mean age = 57.8 ± 10.5, 61.1% female	Healthy control for colorectal cancer *N* = 221 (Italian cohort: 105 samples, including samples from^[92]^; Czech cohort: 36 samples; Validation cohort: 80 samples, including samples from Italy^[82,83]^)	RNA sequencing and alignment using in-house reference based on miRbase v22, 9.8 million average raw sequencing reads, 0.73% aligned to database	220	Pardini *et al.* 2023^[37]^
Italy, age 10-20, 50% female	Healthy control for Autism spectrum disorders *N* = 6	RNA sequencing and alignment using Arena-Idb, 26.2 million average raw sequencing reads, 0.5% aligned to database	28	Chiappori *et al.* 2022^[93]^
Poland, mean age = 36 (range 26-41), 66.7% female	Healthy control for Crohn’s disease *N* = 9	nCounter® Human v2 miRNA Expression Assay (NanoString Technologies)	66	Ambrozkiewicz *et al.* 2020^[94]^ (GEO series GSE144535)

Studies used fecal samples for extraction of total RNA^[33,37,82,83,87,88,91,93]^ or miRNA-enriched RNA^[7,90,94]^. miRNA: MicroRNA; sRNA: small RNA.

**Figure 1:**
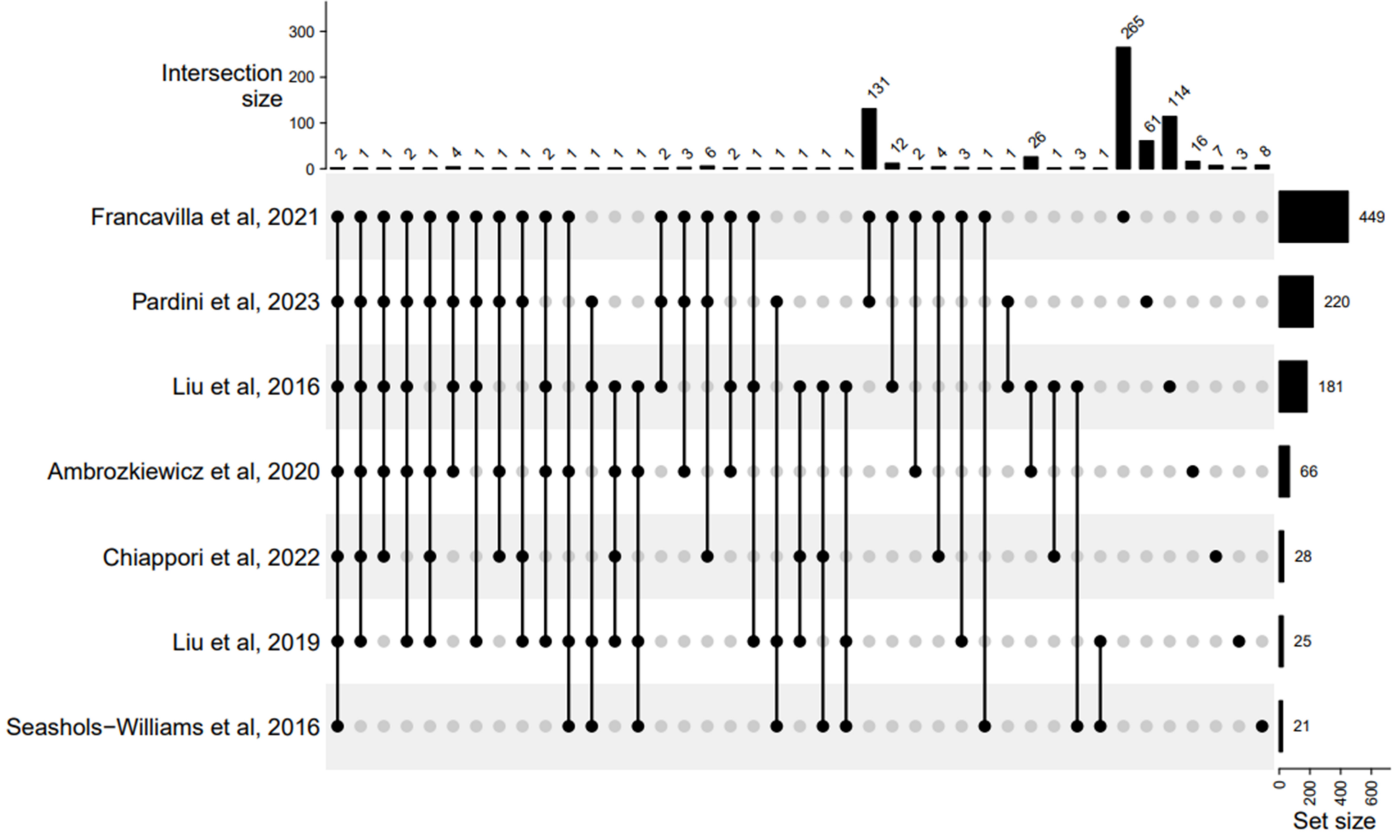
UpSet plot of shared miRNA in healthy human feces from 7 different datasets. Datasets are indicated by the first author’s name and publication year of the corresponding paper. Only miRNAs with abundance above the study-specific threshold were used. The set size represents the total number of miRNAs contained in each dataset. The intersection size represents the number of miRNAs shared exclusively among the dotted datasets listed below (connected black dots); grey dots indicate datasets without these shared miRNAs. Two miRNAs were identified in all 7 datasets. A varying number of miRNAs (1-131) were shared among 2-6 datasets. The plot was created with the ComplexHeatmap R package^[95]^. miRNA: MicroRNA.

The number of fecal miRNAs identified varies largely, with a minimum of 21^[87]^ and a maximum of 449^[33]^. This discrepancy could be explained by technical differences, including the sequencing depth and the miRNA annotation reference library employed for RNA sequencing. MiRNA counts were calculated as the average counts across all healthy samples within a given dataset. MiRNAs with an average count below the detection threshold specified in the corresponding study were excluded from the intersection analysis. The intersection was defined as miRNAs found to be exclusively shared by the datasets of interest (Figure 1, connected black dots).

We found that the intersection of the 7 datasets consists of two shared miRNAs: hsa-miR-21-5p and hsa-miR-1246 [Figure 1]. We noted that the method used for high-throughput miRNA profiling affects the number of miRNAs detected. Studies using NanoString Technologies, which employs unique oligonucleotide tags and a defined set of housekeeping and control miRNAs for threshold calculation, identified a higher number of shared miRNAs (i.e., 42). On the other hand, RNA sequencing offers a more comprehensive assessment, enabling the discovery of new miRNAs, but its results can be affected by the sequencing depth and the threshold chosen. Profiling studies provide information on the relative abundance of different miRNAs, and the shared miRNA hsa-miR-1246 appears to be among the most highly abundant miRNAs across studies. However, NanoString Technologies data indicate that most fecal miRNAs (174 out of 181^[7]^, and 53 out of 66^[94]^) are present at less than 500 average reads. This is particularly important, as quantifying miRNA abundances in feces could have significant clinical applications. Hsa-miR-21-5p plays crucial roles in intestinal homeostasis by regulating gut permeability and immune function^[96]^, while hsa-miR-1246 promotes inflammation through the activation of specific transcription factors^[97]^. Interestingly, elevated fecal levels of hsa-miR-1246 and hsa-miR-21-5p have been observed in colorectal cancer studies^[37,98]^, with hsa-miR-21-5p extensively studied as a potential biomarker^[98]^. In human cancer models, the overexpression of hsa-miR-21-5p regulates key cellular processes involved in cancer development and progression^[99]^. This highlights the potential of fecal miRNAs not only as a diagnostic tool but also as targets for therapeutic interventions.

## CURRENT EVIDENCE ON THE EFFECTS OF MIRNAs ON MEMBERS OF THE MICROBIOTA

In 2016, Liu *et al.* showed that human miRNAs could be uptaken by bacteria^[7]^. Their study visualized synthetic hsa-miR-1226-5p and hsa-miR-515-5p within *Escherichia coli* and *Fusobacterium nucleatum* cells following co-incubation experiments. These revealed the varying capacities of different miRNAs to enter and accumulate within bacterial cells. Liu *et al.* also showed that these two miRNAs supported the growth of these bacteria. Because scrambled negative controls providing similar quantities of the same nucleotides were used, it is possible that these effects on growth are specific and mediated via gene expression regulation. Subsequent studies have investigated the effects of various host and food-derived miRNAs on the growth and gene expression of different bacterial strains [Table 2].

**Table 2:** Current evidence on the effects of host and dietary miRNAs on the growth and gene expression of gut microbiota bacterial representatives and of probiotic strains

**miRNA**	**Effect *in vitro***			**Effect *in vivo***			**Ref.**
	**Bacteria**	**Internalization and growth**	**Targeted gene**	**Condition, model**	**Microbiota outcomes**	**Other outcomes**	
**Host miRNA - microbiota species**							
hsa-miR-876-5p hsa-miR-515-5p hsa-miR-1226-5p hsa-miR-4747-3p hsa-miR-1224-5p hsa-miR-32	*F. nucleatum* ATCC 10953 *E. coli* ATCC 47016	miR-515-5p: enter *F. nucleatum*, colocalize with nucleic acids, ↑ its growth miR-1226-5p: enter *E. coli*, colocalize with nucleic acids, ↑ its growth	miR-515-5p: ↑ *F. nucleatum* 16S/23S rRNA transcripts ratio *E. coli*: miR-1226-5p ↑ yegH. MiR-4747-3p ↑ RNaseP. MiR-1224-5p ↓ rutA. MiR-623 ↓ FucO transcript levels	Mice defective in IEC- or Hopx-expressing cells-specific miRNA (Dicer1ΔIEC, Dicer1ΔHopx) DSS-induced colitis C57BL/6J + miRNA mimics	Dicer1ΔIEC: ↑ microbiota dissimilarity between mice WT + miR-1226-5p: ↑ *E. coli* abundance	Dicer1ΔIEC/Hopx, DSS: ↓ fecal miR abundance, exacerbate colitis symptoms	[7]
hsa-miR-30d-5p	*A. muciniphila* ATCC BAA835	miR-30d enters *A. muciniphila* ↑ ratio *A. muciniphila*/*E. coli*	↑ AMUC_RS06985 and AMUC_RS07700 expression, and ↑ β-galactosidase activity	EAE model, C57BL6J mice EAE + miR-30d mimics Fecal transplant from miR-30d treated EAE mice	No effect on microbiota diversity. Ameliorates EAE in a microbiome-dependent manner. ↑ *A. muciniphila*	Ameliorates EAE (clinical score, demyelination, and axonal loss)	[90]
hsa-miR-4493-5p hsa-miR-3622b-5p	*S. variabile* DSM15176, *O. splanchnicus* DSM20712, *E. coli* K12 DSM498	No effect compared to vehicle or scramble	-	-	-	-	[100]
mmu-miR-200-3p mmu-miR-200b-5p mmu-miR-181b-5p mmu-miR-28b-3p	Mouse fecal microbiota (control and colitis) *E. coli* ATCC 29522	miR-200b-3p: enter various fecal bacteria, colocalize with nucleic acids, ↑ *Lactobacillus*, *Dubosiella*, ↓ *E. coli* miR-200n-3p: enter *E. coli*, colocalize with nucleic acids, ↓ *E. coli* growth miR-181b-5p: no effect on growth, cannot enter fecal bacteria	-	Acute/Chronic DSS-induced colitis, C57BL/6J mice/Wistar rats DSS + miRNA mimics DSS + control/colitis fecal BMVs	miR-200-3p: restore DSS-induced microbiota changes. miR-181b-5p: no protective effects Control-BMV or colitis-BMV + miR-200b-3p: restore microbiota composition to pre-DSS-treated states	DSS + miR-200b-3p/miR-181b-5p: alleviate DSS disease severity DSS + miR-181b-5p: ↑ CD206 and M2 macrophage level Control-BMV or colitis-BMV + miR-200b-3p: restore intestinal barrier	[66]
**Host miRNA - probiotic and other strains**							
hsa-miR-21-5p	*L. reuteri* DSM17938 *L. reuteri* ATCC PTA6475	↓ both *L. reuteri* strains growth	-	Bile duct ligation (BDL), C57BL/6NCrl, and miR-21KO mice WT/miR-21KO co-housing	miR-21KO: prevent BDL-induced dysbiosis ↑ *Lactobacillus spp.* abundance Co-housing: similar relative abundance of *Lactobacillus*; ↓ in KO after 1-month isolation	miR-21KO + BDL: ↓ liver damage and small intestine permeabilization	[101]
mmu-miR-142a-3p mmu-miR-223-5p mmu-miR-142b mmu-miR-146b-5p	*L. reuteri* ATCC23272 *L. johnsonii* ATCC 33200	miR-142a-3p: ↑ *L. reuteri*, no effect on *L. johnsonii*	miR-142a-3p: ↑ *L. reuteri* primase and polymerase I expression	DSS-induced colitis, C57BL/6 mice DSS + miR-142a-3p mimics DSS + miR-142a-3p treated mice FMT	miR-142a-3p: Affect β-diversity, ↑ *L. reuteri* relative abundance, ↑ fecal reuterin	miR-142a-3p: Alleviate DSS disease severity (weight loss, DAI, colon bleeding and swelling)	[102]
mmu-miR-155 mmu-let-7g	*L. gasseri* ATCC 33323 *E. coli*	miR-155, let-7g: enter *E. coli* miR-155: ↓ *L. gasseri* let-7g: no effects	-	Ovariectomy (OVX), C57BL/6J mice miR-155, cel-miR-54, or miR-155 antagonist microspheres	miR-155 microspheres: ↓ *Lactobacillus*, miR-155 detectable in cecal bacteria miR-155 antagonist microspheres: restore *Lactobacillus* level	OVX: ↑ miR-155 and let-7g in feces and intestine miR-155 antagonist microsphere: no effect on fecal miR-155 level. Protect OVX-induced cardiac effects	[103]
hsa-miR-7704 hsa-miR-6127 hsa-miR-4788 hsa-miR-4443 hsa-miR-4740-3p hsa-miR-320e	*B. longum* JCM1217, *E. coli* K-12 MG1655	miR-7704 enters *B. longum* but not *E. coli* miR-7704: change patient microbiota structure and diversity *in vitro*, ↓ *B. longum* relative abundance	miR-7704 pre-treated *B. longum*: ↓ adherence to HT-29 cells ↓ proB, ↑ BLLJ_RS08400 relative expression, ↓ proline levels	HE model, C57BL/6J mice HE + miRNA mimics FMT miR-7704- treated mice	HE patient/mice: ↓ *Bifidobacterium* (*B. longum* and *B. pseudocatenulatum*)	HE + miR-7704: ↑ mortality and neuroinflammation	[104]
**Diet miRNA - microbiota and other species**							
mmu-miR-375 (Packaged in ginger-derived nanoparticle)	-	-	-	Obesity (HFD), C57BL/6 mice HFD + GDNP WT + GDNP packaged with miR-375 (nano-miR375) WT + fecal exosome of HFD mice + nano-miR375	Labeled-IEC exosomes are taken up by 26.5% of gut bacteria GDNP-mediated induction of miR-375 in HFD mice: ↓ *E. coli tnaA* gene expression	GDNP: ↑ miR-375 expression and release in exosome nano-miR375: ↓ AhR expression in small intestine tissue, can be transported to the liver and be taken up by hepatocytes	[105]
bol-miR-159 (Broccoli)	*Bacillus. sp.* ATCC21591 *R. eutropha* CGMCC1.3907 *W. paramesenteroides* ATCC33313	miR159 enter the 3 bacteria and accumulate in it Limited entry of the scramble ↓ *Bacillus. sp.* No effect on *W. confusa* ↑ *W. paramesenteroides* and *R. eutropha*	*celC* gene in *Bacillus*, *rnY* gene in *Weissella*, and *phaZ2* gene in *Ralstonia*	Healthy, BALB/c mice miRNA mimic gavage	↑ the diversity of gut microbiota and affect the β-diversity ↑ *Proteobacteria*, ↓ *Firmicutes/Bacteroidetes* ratio ↑ *Weissella*, *Bacteroides*, *Bifidobacterium*, *Ralstonia*, *Blautia*. ↓ *Bacillus*	No pathological lesions or inflammatory responses	[106]
peu-MIR2916-p5 peu-MIR2916-p3 (Garlic)	Mouse fecal microbiota *B. thetaiotaomicron* VPI-5482 ATCC 29148	GELNs are taken up by gut microbes, and colocalize with *B. thetaiotaomicron* peu-MIR2916-p3: ↑ *B. thetaiotaomicron* peu-MIR2916-p5: no effect	-	Acute/Chronic DSS-induced colitis, C57BL/6J mice DSS + Labelled GELNs (low, medium, high dose)	GELNs: ameliorate DSS-induced loss of richness, restore the *Firmicutes/Bacteroidetes* ratio. Dose-dependent ↑ of *Bacteroides*	GELNs: medium and high doses ameliorate acute and chronic colitis symptoms and alterations of the intestinal barrier	[107]
**Diet miRNA - probiotic and other strains**							
gma-miR396e (*Glycine max*) ath-miR167a-5p (*Arabidopsis thaliana*) mdo-miR-7267-3p (*Monodelphis domestica*) in ginger exosome-like nanoparticles	*L. rhamnosus* LGG ATCC 53103	Ginger ELN, ELN RNA: taken up by LGG Ginger ELN-RNA, gma-miR396e: ↑ LGG	gma-miR396e: ↓ LexA RNA level mdo-miR7267-3p: ↓ ycnE RNA level ath-miR-167a-5p: ↓ SpaC RNA and protein expression	DSS-induced colitis, SPF C57BL/6 mice Healthy mice + ELNs, ELN RNA, or PKH26-labeled ELN C57BL/6 + ath-miR167a treated-LGG + DSS	Ginger ELN, ELN RNA: ↑ *Lactobacillaceae*, ↓ mucosa-associated LGG Ginger ELNs are mainly taken up by *Lactobacillaceae* in the gut	Ginger ELN-RNA: alleviates DSS disease severity in a microbiome-dependent manner; ↑ fecal I3A, ↓ I3AM Ath-miR167a: prevent *L. rhamnosus* from entering the cell	[73]
miR6300 miR482b miR482c-5p (Tartary Buckwheat)	*Lactobacillus rhamnosus* LGG ATCC 53103 *E. coli* ATCC 25922	miR6300: ↑ *E. coli* miR482c and miR482b: no effect on *E. coli* miR3630, miR482b: no effect on LGG	miR3630, miR482b: ↑ overall SCFA production	Healthy, C57BL/6 mice Gavage labeled- TBDNs	TBDNs: ↑ diversity of fecal microorganisms and ↑ the SCFA levels	TBDNs detected in the liver and colon	[108]
cal-miR2911 (cauliflower)	*B. adolescentis* ATCC 15703 *Bacillus sp.* ATCC 21591 *L. casei* ATCC 393	miR2911 enter *B. adolescentis* ↑ *B. adolescentis.* No effect on *Bacillus sp.* or *L. casei*	ATP synthase gene	Healthy, BALB/c SPF mice miR2911 gavage	No effect on *α*-diversity. ↑ *Bifidobacterium* relative abundance, ↑ *Eggerthellaceae*	miR2911 concentration in the intestine ↓ to 1 pM after two hours, remained constant until 8 h	[109]

-: Not assessed. miRNAs: microRNAs; yegH: inner membrane protein, RNaseP: ribonuclease P, rutA: pyrimidine monooxygenase, FucO: lactaldehyde reductase, IEC: intestinal epithelial cells, Hopx: homeodomain only protein, DSS: dextran sulfate sodium, WT: wild type, KO: knockout, EAE: experimental autoimmune encephalitis, proB: glutamate 5-kinase, BLLJ_RS08400: NAD+/NADH kinase, tnaA: tryptophanase, AhR: aryl hydrocarbon receptor, BMV: bacterial membrane vesicles, DAI: disease activity index, FMT: fecal microbiota transplant, HE: hepatic encephalopathy, HFD: high-fat diet, GDNP: ginger-derived nanoparticles, celC: endoglucanase, rnY: ribonuclease, phaZ2: intracellular PHB depolymerase, GELNs: garlic exosome-like nanoparticles, ELN: exosome-like nanoparticles, LexA: transcriptional repressor, ycnE: monooxygenase, SpaC: pilus subunit, I3A: indole-3-carboxaldehyde; I3AM: indole-3 acetamide, TBDNs: tartary buckwheat-derived nanovesicles, SCFA: short-chain fatty acids.

Overall, host miRNAs increase or decrease the growth of Gram-positive and Gram-negative bacteria found in the mouse and human fecal microbiota. This has been demonstrated both *in vitro*^[7,66,90]^ and *in vivo* using rodent models of autoimmune encephalomyelitis^[90]^ and colitis^[7,66]^. However, some miRNAs appeared to have no discernible effect on the growth of gut microbiota members. This could be due to their incapacity to enter bacteria, as further discussed below. For instance, Shen *et al.* found that mmu-miR-200b-3p could enter various fecal bacteria, affecting fecal microbiota composition (increased *Lactobacillus* and *Dubosiella* abundance, decreased *Escherichia coli* abundance), while mmu-miR-181b-5p could not enter fecal bacteria and had no effects on *Escherichia coli* growth or overall microbiota composition^[66]^. Studies have also investigated the effects of host miRNAs on known probiotic species such as *Limosilactobacillus reuteri*, *Lactobacillus johnsonii*, *Lactobacillus gasseri*, and *Bifidobacterium longum.* These studies have shown both enhancement and diminishment of growth *in vitro* and *in vivo* rodent models of colitis^[102]^, hepatic encephalopathy^[104]^, ovariectomy^[103]^, or specific miRNA knockout^[101]^. Again, some host miRNAs exhibited no effects, while others had effects only on certain bacterial species or genera. For example, hsa-miR-142a-3p promoted the growth of *Limosilactobacillus reuteri* but not *Lactobacillus johnsonii in vitro* and in a DSS-induced colitis model^[102]^. There is currently limited knowledge of the effects of miRNA on different strains within the same species. Host miRNAs appeared to be able to increase or decrease bacterial transcripts of genes involved in energy production (NAD+/NADH kinase), nutrient degradation (pyrimidine, fucose, lactose degradation), and DNA or RNA synthesis [ribonuclease (RNase), primase, polymerase]. This has been suggested to explain miRNAs’ overall effects on bacterial growth. These studies investigated the effects of free host miRNA using either purified fecal miRNA or synthesized double-strand or single-strand miRNA mimics. Bacteria were mostly co-cultured with miRNAs in different concentrations ranging from 0.5 to 20 μM, and growth was assessed using growth curves^[7,102,108]^ or culturing^[101]^.

To our knowledge, no studies have explored the impact of host exosomal fecal miRNAs, despite suggestions that IEC-derived exosomes might be taken up by more than one-quarter of gut bacteria^[105]^. The effect of diet-originating miRNA on bacterial members of the gut microbiota or probiotics further expands our understanding of this process. Studies have predominantly explored the uptake of plant miRNAs through exosomes or small vesicles, either directly purified from plant products or synthesized and packaged with plant miRNA mimics. Plant miRNAs have been shown to both increase and decrease the growth of Gram-positive or Gram-negative bacteria members of the gut microbiota *in vitro* or *in vivo*, using a healthy rodent model of miRNA administration^[106]^, obesity^[105]^, or colitis^[107]^. The effects of dietary miRNAs appeared to be sequence-specific and consequential to their entry into bacteria cells. For example, Xu *et al.* found that bol-miR-159 could enter and accumulate in *Bacillus spp*, *Ralstonia eutropha*, and *Weissella paramesenteroides* to affect their growth, while its scrambled counterpart had limited entry and no growth-modulating effect^[106]^. Studies also reported the effects of plant miRNAs on probiotics such as *Lacticaseibacillus rhamnosus* GG and *Bifidobacterium adolescentis*, reporting an increase in their growth *in vitro* and in healthy^[109]^ or colitis mouse models^[73]^. Similarly to host miRNAs, plant miRNAs have been suggested to target bacterial genes involved in nutrient degradation (tryptophanase, endoglucanase, intracellular PHB depolymerase), energy production (ATP synthase), DNA or RNA synthesis (RNase, transcription repressor), as well as genes involved in bacterial adhesion (pilus protein) or metabolite production (short-chain fatty acids).

It is well known that members of the gut microbiota interact with each other to maintain homeostasis and a health-compatible microbiota profile^[110,111]^. Therefore, it is likely that miRNA alteration of the growth of selected microbiota members may indirectly affect other members of the ecosystem, resulting in a change in the gut microbiota composition. This change in composition is indeed observed in multiple studies, where the fecal microbiota β-diversity is affected *in vivo* by miRNA administration^[7,102,106-108]^. Causal relationships need to be confirmed. Finally, *in vitro* fermentation models also reported the effects of host hsa-miR-200b-3p and hsa-miR-7704 on the structure and composition of the mouse fecal microbiota during colitis^[66]^ and chronic hepatitis B^[104]^. The syntrophic interactions among members of the gut microbiota add another layer of complexity to the host-microbiota crosstalk, making it challenging to distinguish the direct and indirect effects of miRNAs on bacteria abundances when studying complex communities.

## POTENTIAL MECHANISMS BY WHICH PROKARYOTES MAY UPTAKE FOREIGN MIRNA

Most publications investigating the effect of host- or plant-derived miRNAs on microbiota or probiotic bacteria have shown the ability of bacteria to uptake miRNA. These findings were obtained using either labeled exosome-like nanoparticles^[73,107]^ or “naked” labeled miRNAs^[7,66,90,103,104,106,109]^ in co-incubation experiments with bacteria or complex fecal ecosystems. Such evidence suggests that multiple miRNA-uptake mechanisms may exist in bacteria, allowing the entry of both encapsulated and free miRNAs.

Since the discovery of bacterial production of membrane-bound secretory vehicles in 1966 in *Escherichia coli*^[112]^, extensive evidence has shown that both Gram-negative and Gram-positive bacteria release bacterial membrane vesicles (MVs). MVs, ranging in size from 10-400 nm, are spherical vesicles with a bilayer lipid membrane structure formed through complex biogenesis mechanisms or following cell lysis^[113]^. These vesicles contain a variety of functional molecules, including proteins^[114]^, lipids^[115]^, DNA^[116]^, and various RNA types, such as mRNA, tRNA, rRNA, sRNA, and miRNA-size small RNA (msRNA)^[117-119]^. MVs play important roles in bacterial survival and colonization, such as nutrient binding, waste removal, biofilm formation, adsorption of detrimental agents (antibiotics, phages), gene transfer, bacterial killing, and quorum sensing, thereby facilitating bacteria-bacteria communication in their environment^[120]^. The recent focus on MVs lies in their role in host-bacteria trans-kingdom communication and their impact on host health and diseases. Notably, bacterial sRNA and msRNA carried by MVs have been shown to enter host cells and use miRNA-like regulatory mechanisms to modulate the host immune system^[121,122]^. Since communication through EVs is an evolutionarily conserved process, it is conceivable that an opposite mechanism may exist, where bacteria uptake eukaryotic miRNA-containing EVs using a similar mechanism.

The internalization of bacterial MVs into eukaryotic cells occurs through five different mechanisms: macropinocytosis, clathrin-mediated endocytosis, caveolin-mediated endocytosis, lipid raft-mediated endocytosis, or direct membrane fusion^[123]^. In prokaryotes, the fusion of MVs with bacterial cells remains less described. MVs originating from *Bacillus subtilis* have been observed to attach and fuse to the outer membrane of their parent bacteria^[124]^. Moreover, bacterial MVs have demonstrated the capacity to transport antibacterial compounds to other bacterial species. For example, MVs derived from the probiotic *Lactobacillus acidophilus* ATCC 53544 can fuse with *Lactobacillus delbrueckii* subsp. *lactis* ATCC 15808 to deliver bacteriocin^[125]^. Similarly, *Streptomyces* MVs can fuse with both Gram-positive (*Streptococcus aureus*) and -negative (*Klebsiella pneumoniae*) bacteria, as well as eukaryotic microbial cells (*Candida albicans*, *Cryptococcus neoformans*) to deliver antimicrobial compounds^[126]^. The recognition of MVs by bacteria is thought to be mediated by LPS and tethers, maintaining MVs at a small distance from the cell (10-20 nm)^[127,128]^. This MV cargo delivery mechanism is suggested to be specific, potentially involving ligand-receptor interactions^[129]^ and influenced by the physicochemical characteristics of both bacterial and EV membranes^[120]^. Therefore, MVs could be secreted by bacteria to target specific cells or could be specifically recruited by bacteria to favor their own growth. This leads us to speculate that host EVs containing miRNAs might also target specific bacterial cells and fuse through similar mechanisms. However, to our knowledge, the processes of host EV internalization by prokaryotes have not yet been described.

Although multiple studies have demonstrated the entry of mature, naked miRNAs into bacteria^[7,66,90,103,104,106,109]^, the mechanisms underlying this phenomenon remain largely unexplored. In addition, specific miRNAs seem to only enter specific bacteria^[7,102,106-109]^, suggesting that this mechanism may be selective or sequence-dependent. It has been first thought that naked miRNAs might be translocated into host-derived microvesicles in the gastrointestinal tract, to further be uptaken by bacteria through fusion^[90]^. However, a rationale exists for naked miRNA uptake since miRNAs have been shown to enter human mitochondria from the cytoplasm and promote the translation of mitochondrial transcripts^[130]^. Although the exact mechanism of miRNA entry into mitochondria remains unclear as well, it may involve the AGO2, the polynucleotide phosphorylase (PNPase), and the voltage-dependent anion channel (VDAC)^[131]^. Mitochondrial porins, like VDAC, share functional and structural similarities with bacterial porins, suggesting that similar membrane structures in bacteria could facilitate miRNA uptake^[132,133]^. PNPase, an evolutionarily conserved enzyme known for its role in RNA turnover in bacteria, has also been shown to participate in sRNA-mediated gene regulation by promoting sRNA stability^[134]^. These observations suggest that these proteins, due to their structural and functional similarities with their eukaryotic counterparts, may play a crucial role in the uptake and regulation of miRNA in bacteria. However, further research is needed to elucidate these mechanisms and determine if bacterial porins or PNPase contribute to selective miRNA uptake. Finally, it was also suggested that a mechanism similar to the horizontal gene transfer involving a DNA receptor^[135,136]^ may exist for RNA, although no such receptor has yet been identified.

In summary, studies have demonstrated that bacteria can internalize miRNAs using both labeled exosome-like nanoparticles and naked miRNAs, suggesting diverse uptake pathways may exist. The internalization of MVs into eukaryotic cells occurs through various endocytic pathways, and similar mechanisms might be involved in the uptake of host-derived EVs by prokaryotes. Additionally, the selective and sequence-dependent uptake of naked miRNAs by bacteria indicates the potential involvement of specific bacterial porins or other membrane proteins. While the exact mechanisms remain largely unexplored, these first findings highlight the specificity of miRNA uptake in bacteria.

## SRNA REGULATION OF GENE EXPRESSION IN PROKARYOTES

**Figure 2:**
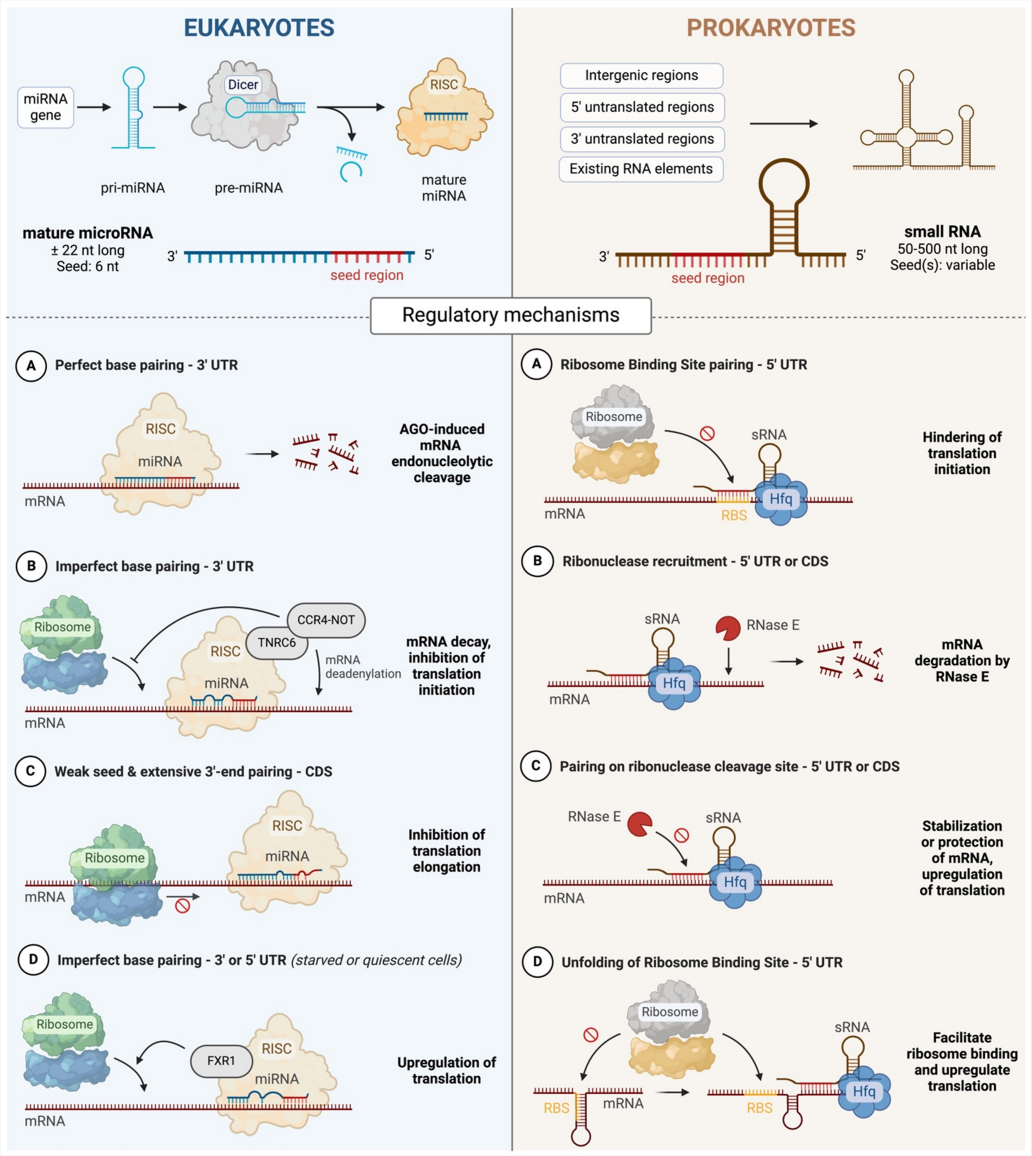
Structure and mechanisms of action of eukaryotic miRNA and prokaryotic sRNA. Left panel: miRNA genes are transcribed into pri-miRNA and undergo processes to liberate pre-miRNA that are exported to the cytoplasm. Pre-miRNAs are then further cleaved by the enzyme Dicer. The miRNA duplex is then loaded into an AGO, to form the RISC after expulsion of the passenger strand. A: A perfect base pairing between a miRNA and its target 3’ UTR sequence triggers an endonucleolytic mRNA cleavage induced by AGO. B: The formation of a partial duplex (imperfect base pairing) in the 3’ UTR destabilizes mRNA through the recruitment of TNRC6 proteins and the CCR4-NOT complexes, which induce mRNA deadenylation. CCR4-NOT further inhibits translation, notably through the recruitment of a helicase. C: An extensive 3’-end pairing on the CDS, without a strong seed pairing, induces ribosome stalling and, therefore, inhibits mRNA translation. D: Under specific conditions, such as amino-acid starvation or cell cycle arrest, miRNA binding to the 3’ or 5’ UTR can upregulate translation in an unknown process involving the miRNA binding proteins AGO2 and FXR1. Right panel: Prokaryotic sRNAs originate from intergenic regions, 5’ or 3’ UTRs, or are derived from existing RNA elements. The sRNA regulatory mechanisms described are limited to Hfq-mediated pairing, although other chaperone proteins and Hfq-independent mechanisms exist. A: sRNA pairing to the RBS, including the SD sequence or the AUG start codon, limits ribosome assembly and hinders translation initiation. B: sRNA pairing in the 5’ UTR or CDS recruits RNase E and induces mRNA degradation. C: On the 5’ UTR or CDS, sRNA can stabilize mRNA secondary structure or bind to RNase cleavage sites and protect RNase-sensitive mRNA from degradation, thereby upregulating translation. D: sRNA binding at the 5’ UTR can help unfolding mRNA to reveal sequestered RBS and facilitate ribosome binding and translation. Created with BioRender.com. miRNA: MicroRNA; sRNA: small RNA; pri-miRNA: primary miRNA; pre-miRNA: precursor miRNA; AGO: Argonaute protein; RISC: RNA-induced silencing complex; UTR: untranslated region; CDS: coding sequence; RBS: ribosome binding site; SD: Shine-Dalgarno; RNase: ribonuclease.

Although no prokaryotic miRNAs have been identified so far, bacteria and archaea express various types of sRNA that regulate gene expression. These prokaryotic sRNAs range from 50 to 500 nucleotides in length. They originate from intergenic regions^[137]^, 5’ or 3’ UTRs^[138]^, or are processed from existing RNA elements like tRNA^[139]^ or rRNA^[140]^ [Figure 2]. Although prokaryotic sRNAs are longer than eukaryotic miRNAs, the length of their base-pairing sites could be independent of their overall size. Most sRNAs contain a seed region of less than 20 nucleotides^[141]^. In 2012, Lee *et al.* were the first to report the existence of a msRNA of 15-28 nucleotides^[142]^ in *E. coli.* This msRNA, similarly to eukaryotic miRNAs, can be derived from hairpin structures^[143]^. Since then, other researchers have identified msRNA in bacteria with pathogenic potential^[119,144,145]^. For example, Furuse *et al.* used deep sequencing to identify a miRNA-like compound expressed by *Mycobacterium marinum.* This compound can bind to the RISC complex and act on an artificial reporter gene with a perfectly complementary target site^[145]^. However, this sRNA is not found in the absence of infectable cells, suggesting that it requires the host cellular machinery for its biosynthesis^[145]^.

In contrast to eukaryotic miRNAs, prokaryotic sRNAs exhibit diverse mechanisms of action in gene regulation. They can bind to their target mRNAs in either a perfect or imperfect manner, affecting mRNA translation or stability. sRNAs typically bind to the 5’ UTR of mRNA. This includes the Shine-Dalgarno (SD) sequence, a ribosome binding site (RBS) in prokaryote mRNA located upstream of the start codon^[146]^. By binding to this region, sRNAs can hinder translation initiation by limiting ribosome assembly^[147]^. Prokaryotic sRNAs can also bind to the CDS of mRNA^[148]^ through interactions often mediated by chaperone proteins. These chaperones protect sRNA from degradation and facilitate its paring to target mRNA^[149,150]^. Hfq (Host Factor required for the replication of bacteriophage Qβ, as identified in *E. coli*^[151]^) is a key chaperone protein and helps recruit RNase E to induce rapid degradation of mRNA^[152]^. Not all bacteria possess Hfq, and other helper proteins, such as ProQ^[153]^, can act as alternative chaperones. The existence of additional proteins assisting sRNA function remains to be further investigated. Some sRNAs also function independently of chaperones. For example, they can interact directly with specific regions in mRNA, such as stem-loop structures or C-A-rich regions, which act as translational enhancers. These interactions can, therefore, modulate ribosome binding and translation^[141]^. Finally, prokaryotic sRNAs can also act at the transcription level. In this case, sRNAs influence whether transcription continues by modulating the binding of Rho, a termination factor, to mRNA^[154,155]^. This modulation either inhibits or promotes transcription termination.

This versatility allows prokaryotes to quickly adapt to environmental changes and stress conditions. For instance, studies in the archaeon *Haloferax volcanii* illustrate how exposure to oxidative stress alters the production of sRNAs; these include responsive antisense sRNA that can align to various regions of mRNA, such as the 5’ UTR, 3’ UTR, and CDS^[156]^. Under alkaline stress, however, the organism expresses a valine tRNA-derived fragment that potentially binds to ribosomes, competing with mRNA and reducing global translation; this contrasts with the specific gene regulation typically associated with miRNA^[157]^. Additionally, sRNAs are involved in bacterial sugar metabolism, including the uptake and degradation of galactose, glucose, and amino sugars^[158]^, suggesting they could have a direct impact on bacterial growth. sRNAs are also involved in bacterial communication with their surrounding environment. For example, the sRNA MicA has been found to regulate the porin protein OmpA that forms channels in the outer membrane of *E. coli*^[159]^, and to induce the production of outer MVs^[160]^. Recent findings also suggest that bacteria may secrete sRNAs along with their chaperone proteins through outer MVs. Indeed, the helper protein Hfq, potentially bound to sRNA, has been detected in EVs of *E. coli*^[161]^.

Research on the effects of host or diet-derived miRNAs on gut microbiota members reveals parallels with bacterial sRNA mechanisms, where host and plant miRNAs can either increase (AMUC_RS06985: β-galactosidase; RNaseP: ribonuclease P; LREU_RS03575: DNA primase; polA: DNA polymerase I) or decrease (BLLJ_RS08400: NAD+/NADH kinase; RutA: pyrimidine monooxygenase; FucO: Lactaldehyde reductase; tnaA: tryptophanase; LexA: transcriptional repressor; proB: glutamate 5-kinase; spaC: pilus adhesin; ycnE: putative monooxygenase) mRNA transcript abundance [Table 2]. This suggests that eukaryotic miRNAs may act on bacterial gene expression post-transcriptionally. They may bind perfectly or imperfectly to complementary sequences at the 5’UTR or within the CDS of bacterial mRNAs. Helper proteins might facilitate these interactions, which can either inhibit or increase mRNA translation, depending on the type of binding and its location. Additionally, miRNAs may influence bacterial mRNA stability, resulting in either its degradation or stabilization. In bacteria, the processes of transcription and translation are often concurrent, as ribosomes can bind and rapidly cover newly synthesized mRNA^[162]^. One hypothesis explaining how sRNA may bind to mRNA in this context relies on the strength of the SD sequence, proposing that sRNA could bind more easily to mRNAs with a “weak” SD sequence, as weaker sequences would not attract and bind ribosomes as strongly^[148]^. Following this idea, eukaryotic miRNAs may only base pair with mRNA with a “weak” SD sequence. Such a sequence would slow down the ribosome assembly rate, making it easier for miRNAs to bind and interact with mRNA. Additionally, it is known that the effectiveness of sRNAs in repressing target genes depends on the balance between the rates of sRNA synthesis (i.e., sRNA concentration) and mRNA synthesis^[163]^. When the transcription rate of sRNA is higher than that of the target mRNA, gene expression is silenced. Conversely, if the sRNA transcription rate is lower, the unbound mRNA can be translated into proteins. More specifically, sRNA-mediated regulation is thought to exhibit a threshold-linear response determined by their target synthesis rate above which sRNAs have little to no effect^[163]^. This concept aligns with flow cytometry experiments that report a dynamic accumulation of miRNA in bacteria^[7,73,104,106,107,109]^. These experiments suggest that miRNA may accumulate inside the cell to reach the minimal concentration needed to affect bacterial gene expression. Therefore, the sRNA concentration relative to its target may explain why miRNA effects are observed in studies using miRNA concentrations ranging from 5 nM^[103]^ to 20 mM^[108]^. This range observed *in vitro* may indicate that miRNAs must reach certain levels to exert their regulatory effects on their bacterial target gene, and these levels may differ for various miRNA-gene pairs. Finally, previous research reports a colocalization of miRNA with bacterial DNA near the nuclear regions of bacteria and suggests that miRNA may act directly at the DNA level^[7,66,106]^. Even though no such mechanism has been demonstrated, similarly to sRNA, miRNA may affect gene expression at the transcription level, for example, through transcription termination. Interestingly, the RNA-binding protein Hfq has been shown to interact with double- and single-stranded DNA^[164,165]^, suggesting an interaction with DNA might be possible. Future studies will need to investigate with more precision the localization of miRNA and their targets in bacteria cells to better understand their mechanisms, especially since the localization of regulatory RNA and mRNA is now recognized as a critical factor for gene expression regulation in prokaryotes^[166]^.

## METHODS TO IDENTIFY POTENTIAL PROKARYOTIC GENE TARGETS OF HOST AND DIET-DERIVED MIRNAS

MiRNA sequences and annotations, including pre-miRNA stem-loop structure and experimental verification, are available through miRNA repositories such as the widely used miRBase (https://www.mirbase.org/)^[9]^. Eukaryotic gene targets can be predicted through various algorithms that consider a combination of features, such as seed sequence pairing, duplex stability, evolutionary conservation, targeted site accessibility, and the number of target sites^[167]^. An example of a tool that combines all of these features is TargetScan (https://www.targetscan.org/)^[168]^, the most recent release of which utilizes a biochemical model and a convolutional neural network to predict the interaction between miRNA (mostly the seed region) and target mRNA^[169]^. Machine learning prediction tools, such as DeepMirTar^[170]^ or DIANA-microT^[171]^, also exist and can identify potential miRNA targets after being trained on proven miRNA-mRNA interaction datasets^[167]^. However, to date, no tool has yet been developed specifically for *in-silico* prediction of eukaryotic miRNA targets in prokaryotic organisms.

Current literature identifying potential prokaryotic gene targets of host and diet-derived miRNAs relies on sequence-based analysis and RNA thermodynamics. The pioneering study by Liu *et al.* in 2016 aligned bacterial nucleic acid sequences with existing miRNAs using the miRBase searching tool^[7]^. Specifically, Liu *et al.* input the sequences of individual genes, operons, or entire genomes into miRBase and found many miRNAs from various eukaryotic organisms that could potentially target these bacterial sequences based on similarity^[7]^. However, due to the short length of miRNAs, it is highly plausible that most potential matches arise by chance and do not represent a true biological regulatory process. Since then, the Basic Local Alignment Search Tool (BLAST)^[73,90,102,104,105,107]^ appears to be the most commonly used algorithm. BLAST aligns nucleotide sequences of interest with genome sequences from the National Center for Biotechnology Information (NCBI) database, predicting possible base-pairing between miRNA and genes^[172]^. Most previous work has compared bacterial genome sequences to entire mature miRNA sequences^[90,104,104,105]^, while some restricted the alignment to miRNA seed sequences^[102]^ or their reverse complement^[73]^. However, bacterial sRNAs typically exhibit longer seed regions than miRNAs^[173]^, suggesting that the entire miRNA sequence may have to be considered for analysis. Indeed, Liu *et al.* in 2019 confirmed the regulatory effect of miR-30d-5p on *Akkermansia muciniphila* β-galactosidase, showing that nucleotides 2 to 6 of miR-30d-5p were not involved in base pairing^[90]^. It is thus plausible that restricting sequence comparison to the seed sequence may miss potential bacterial gene targets.

Sequence alignment analysis does not account for RNA secondary structure, which is crucial for determining the stability and probability of a miRNA-mRNA duplex formation. To better predict miRNA base-pairing dynamics, studies have assessed the free energy of binding (ΔG) of miRNA-mRNA interactions^[90,104]^. For example, Liu *et al.* used RNAhybrid to confirm the structure of the binding between miR-30d-5p and its potential gene target in *Akkermansia muciniphila*, initially identified by BLAST^[90]^. Other studies^[106,108,109]^ have used miRanda, a miRNA gene target detection tool developed for animals that takes into account sequence, secondary structure, and duplex stability^[174]^. In these methods, the free energy of binding is a crucial criterion: a lower ΔG signifies that more energy is required to break the binding, therefore suggesting stable pairing^[175]^. Studies have selected miRNA targets with the lowest ΔG possible^[90,104]^ or used an arbitrary threshold of ΔG < -10 kcal/mol^[109]^. However, no binding free energy threshold has been established for miRNA-mRNA interactions in prokaryotes or eukaryotes. In addition, while ΔG considers intermolecular pairing, it neglects intramolecular folding and the energy required to open binding sites. Eukaryotic studies have also emphasized the importance of target structural accessibility for miRNA binding^[176,177]^. This suggests that gene target prediction methods may need to account for target accessibility, which can be assessed by determining the total free energy of binding (ΔΔG or ΔG_TOTAL_) using tools like RNAup^[175]^. Horne *et al.* followed this method, using NanoString Technology to profile mouse fecal miRNAs, BLASTn to predict potential bacterial gene targets, and RNAup to calculate the total free energy of binding^[178]^. However, it should be noted that these tools were not initially developed for the prediction of miRNA targets in prokaryotes; therefore, their precision and accuracy in this context remain to be validated.

Confirming miRNA targets in prokaryotes remains challenging. A common approach uses quantitative polymerase chain reaction (qPCR) to determine if the expression level of target genes changes in response to synthetic miRNA, compared to a scramble-sequenced miRNA^[7,73,90,102,104]^. Limitations of this approach include a lack of proof of miRNA binding to specific target mRNAs and overlooking indirect effects on upstream regulatory elements. Other indirect methods include measuring metabolites produced by the encoded protein^[108]^ or observing miRNA effects on bacterial growth^[66,101-104,106,108,109]^. However, these approaches only capture the secondary effects of miRNA-gene modulation and do not validate miRNA-mRNA interactions. The mechanisms of miRNA uptake and regulation in prokaryotes are not well understood, complicating the development of direct confirmation assays and making it challenging to design effective validation experiments or translate *in silico* predictions into biological applications.

MiRNA target confirmation in eukaryotes is more advanced, with well-established techniques such as the luciferase reporter assay, a gold-standard method^[179]^. In this assay, cells are transfected with plasmids containing the miRNA target sequence fused to the 3’ end of a luciferase coding sequence. If the miRNA binds to the target sequence, changes in luminescence can be measured after cell lysis and substrate addition, confirming the interaction. In prokaryotes, similar GFP-based fluorescence assays have been adapted to validate sRNA targets^[180]^. These established methods in eukaryotes and prokaryotes provide valuable insights for adapting target confirmation techniques for bacterial miRNA studies. For instance, developing bacterial-compatible reporter assays or applying high-throughput sequencing-based methods could enhance the accuracy of miRNA target validation in bacteria. With further research into miRNA uptake direct action on mRNA, these approaches could help translate computationally predicted miRNA targets into experimentally confirmed biological phenomena in bacterial systems.

## CONCLUSION

Fecal miRNAs represent a complex and dynamic interface between the host, diet, and gut microbiota. While significant progress has been made in characterizing the fecal miRNome and identifying key miRNAs involved in intestinal homeostasis, many questions remain unanswered. The form in which miRNAs exist in the gut lumen (e.g., exosomal or naked) requires further investigation to better understand their stability and functional relevance. This will contribute to the exploitation of fecal miRNAs in clinical applications as diagnostic biomarkers and therapeutic tools. Moreover, the impact of diet and microbiota composition on the fecal miRNome remains an area of active research. These findings will not only underscore the complexity of miRNA interactions within the gut but also reveal novel mechanisms by which the host can modulate the intestinal microbiota. The human fecal miRNome seems to be individualized. This diversity may partly explain the interindividual variability observed in responses to treatments targeting the microbiota, and that could be modulated via miRNA. Engineered miRNA mimics offer exciting prospects for selectively modulating gut microbiota and enabling personalized interventions in gut health.

Recent evidence suggests that miRNAs can directly interact with gut microbiota by modulating bacterial growth and gene expression. The discovery that both nanoparticle-encapsulated and naked miRNAs can enter bacterial cells may indicate the existence of multiple uptake mechanisms, although the exact pathways remain poorly investigated. Host- and diet-derived miRNAs may influence bacterial growth by regulating genes involved in nutrient degradation and DNA/RNA synthesis. Research is needed to uncover the mechanisms of miRNA entry and their regulatory effects, and it could draw on existing knowledge about prokaryotic sRNAs and eukaryotic miRNA gene regulation. Advancing this field will require the development of tailored computational tools and experimental methodologies, using appropriate controls to assess miRNA direct effects. A comprehensive understanding of the diversity, origin, and functional roles of fecal miRNAs in gut health will require interdisciplinary efforts that integrate microbiology, RNA biology, bioinformatics, and systems biology, to name a few. Studies that combine miRNA profiling with omics data would be needed to investigate the impacts of miRNA regulatory networks on the intestinal microbiota. Bridging these knowledge gaps will help reveal the role of miRNAs in maintaining gut health and explore their potential use in disease prevention and treatment strategies.

## DECLARATIONS

### Copyright

© The Author(s) 2025.
